# Drug interactions may be important risk factors for methotrexate neurotoxicity, particularly in pediatric leukemia patients

**DOI:** 10.1007/s00280-016-3153-0

**Published:** 2016-09-22

**Authors:** Victoria J. Forster, Frederik W. van Delft, Susan F. Baird, Shona Mair, Roderick Skinner, Christina Halsey

**Affiliations:** 1 Paul O’Gorman Building, Northern Institute for Cancer Research, Newcastle University, Framlington Place, Newcastle upon Tyne, NE2 4HH UK; 2 Royal Hospital for Sick Children, Edinburgh, UK; 3 Great North Children’s Hospital, Newcastle upon Tyne, UK; 4 Institute of Cancer Sciences, University of Glasgow, Glasgow, UK

**Keywords:** Neurotoxicity, Hematology, Methotrexate, Nitrous oxide, Leukemia, Toxicity

## Abstract

**Purpose:**

Methotrexate administration is associated with frequent adverse neurological events during treatment for childhood acute lymphoblastic leukemia. Here, we present evidence to support the role of common drug interactions and low vitamin B_12_ levels in potentiating methotrexate neurotoxicity.

**Methods:**

We review the published evidence and highlight key potential drug interactions as well as present clinical evidence of severe methotrexate neurotoxicity in conjunction with nitrous oxide anesthesia and measurements of vitamin B_12_ levels among pediatric leukemia patients during therapy.

**Results:**

We describe a very plausible mechanism for methotrexate neurotoxicity in pediatric leukemia patients involving reduction in methionine and consequential disruption of myelin production. We provide evidence that a number of commonly prescribed drugs in pediatric leukemia management interact with the same folate biosynthetic pathways and/or reduce functional vitamin B_12_ levels and hence are likely to increase the toxicity of methotrexate in these patients. We also present a brief case study supporting out hypothesis that nitrous oxide contributes to methotrexate neurotoxicity and a nutritional study, showing that vitamin B_12_ deficiency is common in pediatric leukemia patients.

**Conclusions:**

Use of nitrous oxide in pediatric leukemia patients at the same time as methotrexate use should be avoided especially as many suitable alternative anesthetic agents exist. Clinicians should consider monitoring levels of vitamin B_12_ in patients suspected of having methotrexate-induced neurotoxic effects.

Adverse neurological events are very common during treatment for pediatric acute lymphoblastic leukemia (ALL) and include seizures, stroke-like syndrome and leukoencephalopathy. Recent trials report neurological adverse events in 4–20 % of patients [1, 2]. Additionally, chronic neurotoxicity is emerging as a worrying late effect [3], and 40–60 % of childhood ALL survivors experience neurocognitive difficulties [4], with methotrexate strongly implicated. Despite toxicities, this mainstay of ALL therapy, given intravenously, orally and intrathecally (IT) is credited with driving down the incidence of central nervous system (CNS) relapse without the need for radiotherapy.

Additionally, although rare, neurotoxicity with similar radiological features of leukoencephalopathy has occasionally been reported following oral methotrexate used in patients with autoimmune and inflammatory disorders [5, 6], where neurotoxicity may appear many years into treatment in patients on a stable dose of methotrexate. Genome wide association studies in childhood ALL patients have failed to conclusively identify any predictive genetic markers for methotrexate neurotoxicity [7]. Neurotoxicity is not directly dose related and does not necessarily occur on first exposure or on re-exposure after a neurological event [8] as may be expected if attributed to genetic vulnerability. This suggests that additional risk factors may be important.

We propose that methotrexate-induced neurotoxicity may be potentiated by common drug interactions, and/or the presence of low vitamin B_12_ (cobalamin) levels, which lead to elevated methotrexate levels in cerebrospinal fluid (CSF) or result in synergistic or additive effects on convergent metabolic pathways. A particularly important and under-appreciated drug interaction may be the concomitant use of inhaled nitrous oxide (N_2_O) and methotrexate, which is common practice in many pediatric hematology centers where lumbar punctures to administer intrathecal (IT) methotrexate are performed under general anesthesia.

A recent case report highlighted the risk of severe neurotoxicity with the combination of N_2_O and methotrexate [9]. Here, we briefly present a second case with multiple acquired risk factors. A 12-year-old girl with acute undifferentiated leukemia received regular IT methotrexate as part of her chemotherapy schedule. All IT therapy was administered under general anesthesia with propofol induction followed by a gaseous mix of N_2_O and oxygen. Other concomitant drugs included the proton-pump inhibitor (PPI) omeprazole. Four days after the 5th dose of IT methotrexate, she was presented with focal seizures rapidly progressing to generalized tonic–clonic seizures, disinhibition, severe agitation and left upper limb (LUL) weakness. MRI (T2/FLAIR and diffusion weighted) imaging on day 2 showed widespread hyperintense subcortical white matter lesions in the frontal and parietal regions with areas of restricted diffusion consistent with leukoencephalopathy. Seizure activity continued for 5 days despite maximal anticonvulsant doses of benzodiazapines and levetiracetam and the radiological changes worsened with increasing cerebral edema and pressure effects requiring dexamethasone. She made a clinical recovery over the next 7 days but with some residual LUL weakness. Serum vitamin B_12_ was measured on recovery (3 weeks from the last IT methotrexate) and was low at 154 ng/L (normal range 200–1100 ng/L).

Methotrexate exerts its anti-leukemic action via inhibition of the enzyme dihydrofolate reductase, ultimately reducing the amount of tetrahydrofolate available for DNA synthesis leading to cell death (summarized in Fig. 1). The reduction in tetrahydrofolate also results in reduced synthesis of methionine from the precursor homocysteine by the enzyme methionine synthase, which requires vitamin B_12_ as a co-factor. Methionine is then converted to S-adenosyl methionine (SAM), a methyl donor which has a critical role in regulating myelin sheath formation and lipid production [10]. This combined with known neuroexcitatory properties of downstream products of homocysteine [11] (Fig. 1) may explain the propensity for CNS side effects in these patients. Indeed, CSF concentrations of SAM were found to be significantly lower in pediatric leukemia patients during methotrexate treatment compared to age-matched controls, whereas levels of myelin basic protein, considered to be a marker of myelin breakdown, were increased [12].

**Fig. 1:**
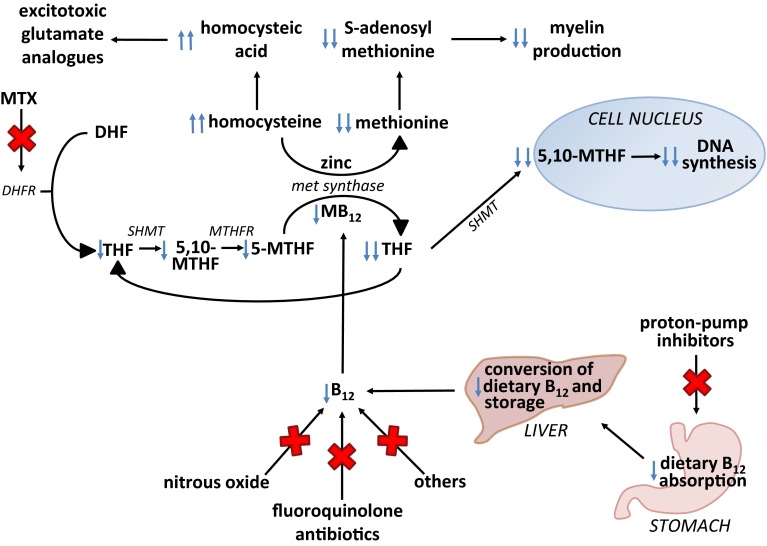
A summary of the biochemical reactions involving folate and vitamin B_12_ inside an oligodendrocyte and proposed inhibition of myelin production by co-administration of methotrexate (MTX) and drugs affecting vitamin B_12_. Abbreviations: *5-MTHF* (5-methyltetrahydrofolate, levomefolic acid), *MB*_*12*_ (methyl B_12_), *THF* (tetrahydrofolate, tetrahydrofolic acid), *5,10-MTHF* (5,10-methylene THF), *DHF* (dihydrofolate, dihydrofolic acid), *DHFR* (dihydrofolate reductase), *MB*_*12*_ (methyl-vitamin B_12_), *MTHFR* (methylenetetrafolate reductase), *MTX* (methotrexate), *met synthase* (methionine synthase), *SHMT* (serine hydroxyl-methyltransferase). MTHF participates in the production of methionine from homocysteine by methionine synthase, catalyzed by MB_12_ and zinc, creating THF and methionine. THF participates in the production of purines and pyrimidines for DNA synthesis. Methionine is a vital amino acid involved in myelin production via its conversion to S-adenosyl methionine (SAM). SAM is involved in the methylation of many proteins and intermediates ultimately involved in myelin production, such as phosphatidylcholine, which is important in the production of sphingomyelin, a major component of the myelin sheath. Homocysteine can be converted to homocysteic acid and homocysteine sulfinic acid which are excitotoxic glutamate analogues acting at the N-methyl-d-aspartate (NMDA) receptor, which may be a factor in acute methotrexate-induced neurotoxicity. Methotrexate inhibits the function of DHFR, preventing the conversion of DHF to MTHF. Active vitamin B_12_ contains reduced cobalt (Co^+^), but nitrous oxide (N_2_O) produces irreversible oxidation to Co^++^ and Co^+++^, rendering vitamin B_12_ inactive. Any simultaneous compromise of folate and vitamin B_12_ via co-administration of methotrexate and agents known to deplete active vitamin B_12_, such as N_2_O could result in increased homocysteine and reduced methionine levels both of which may contribute to the neurotoxic effects of methotrexate treatment. Other as yet unidentified compounds may also reduce bioavailable vitamin B_12_ levels. Blue arrows indicate proposed increase or reduction in various relevant pathway metabolites

Methionine depletion and accumulation of homocysteine may be potentiated by the co-administration of other medications via two mechanisms; (1) a direct drug interaction leading to increased methotrexate plasma (and/or CSF) concentrations or (2) interference with the same metabolic pathways as methotrexate. Examples of (1) include fluoroquinolone antibiotics, piperacillin (the most commonly prescribed antibiotic for episodes of febrile neutropenia during pediatric leukemia treatment in the UK) and proton-pump inhibitors (PPIs). The latter delay plasma elimination of methotrexate leading to renal and liver toxicity [13]. A number of drugs interfere with (2), mainly via depletion of functional vitamin B_12_ (Fig. 1)—as exemplified by nitrous oxide [14]. In addition, PPIs may reduce the bioavailability of dietary vitamin B_12_ [15] and antimetabolites such as 6-mercaptopurine may cause B_12_ malabsorption secondary to enteropathy. Indeed, a small pilot study in our local institution recorded low vitamin B_12_ levels during treatment in 4/19 pediatric patients (21 %) with ALL. Three out of four patients where levels were low experienced severe gastrointestinal enteropathy and 1 patient experienced a severe neurological event. In addition, the same study revealed that 9/17 of the same cohort tested for zinc had clinically low levels at some point during their treatment. Zinc is also a critical co-factor for methionine synthase (Fig. 1); hence, low levels may also contribute to the disruption of this pathway by methotrexate treatment.

The evidence presented above suggests that low B_12_ levels, recent general anesthesia with nitrous oxide or introduction of additional drugs interacting with the same pathways, may be responsible for the idiosyncratic occurrence of methotrexate neurotoxicity in pediatric leukemia patients undergoing treatment with high doses. We aim to raise awareness globally of this potential interaction and particularly ensure that nitrous oxide anesthesia is avoided in all patients on methotrexate as advised by the British National Formulary [16].

In the era where personalized medicine integrated with genomic approaches is seen as the ultimate goal, it is important not to overlook ‘old-fashioned’ drug interactions, especially if such risks can easily be minimized by a change in drug scheduling or use of suitable alternative agents.

## References

[1] Vora A, Goulden N, Wade R (2013). Treatment reduction for children and young adults with low-risk acute lymphoblastic leukaemia defined by minimal residual disease (UKALL 2003): a randomised controlled trial. Lancet Oncol.

[2] Bhojwani D, Sabin ND, Pei D (2014). Methotrexate-induced neurotoxicity and leukoencephalopathy in childhood acute lymphoblastic leukemia. J Clin Oncol.

[3] Schuitema I, Deprez S, Van Hecke W (2013). Accelerated aging, decreased white matter integrity, and associated neuropsychological dysfunction 25 years after pediatric lymphoid malignancies. J Clin Oncol.

[4] Van der Plas E, Nieman BJ, Butcher DT, Hitzler JK, Weksberg R, Ito S, Schachar R (2015). Neurocognitive late effects of chemotheapy in survivors of acute lymphoblastic leukemia: focus on methotrexate. J Can Acad Child Adolesc Psychiatry.

[5] Raghavendra S, Nair MD, Chemmanam T, Krishnamoorthy T, Radhakrishnan VV, Kuruvilla A (2007). Disseminated necrotizing leukoencephalopathy following low-dose oral methotrexate. Eur J Neurol.

[6] Gonzazlez-Suarez I, Aquilar-Amat MJ, Triqueros M, Borobia AM, Cruz A, Arpa J (2014). Leukoencephalopathy due to oral methotrexate. Cerebellum.

[7] Radtke S, Zolk O, Renner B (2013). Germline genetic variations in methotrexate candidate genes are associated with pharmacokinetics, toxicity and outcome in childhood acute lymphoblastic leukemia. Blood.

[8] Badke C, Fleming A, Iqbal A, Khilji O, Parhas S, Weinstein J, Morgan E, Hiiya N (2015). Rechallenging with intrathecal methotrexate after developing subacute neurotoxicity in children with hematologic malignancies. Pediatr Blood Cancer.

[9] Lobel U, Trah J, Escherich G (2015). Severe neurotoxicity following intrathecal methotrexate with nitrous oxide sedation in a child with acute lymphoblastic leukemia. Pediatr Blood Cancer.

[10] Chamberlin ME, Ubagai T, Mudd SH, Wilson WG, Leonard JV, Chou JY (1996). Demyelination of the brain is associated with methionine adenosyltransferase I/III deficiency. J Clin Invest.

[11] Vijayanathan V, Gulinello M, Ali N, Cole PD (2011). Persistent cognitive deficits, induced by intrathecal methotrexate, are associated with elevated CSF concentrations of excitotoxic glutamate analogs and can be reversed by an NMDA antagonist. Behav Brain Res.

[12] Surtees R, Clelland J, Hann I (1998). Demyelination and single-carbon transfer pathway metabolites during the treatment of acute lymphoblastic leukemia: CSF studies. J Clin Oncol.

[13] Suzuki K, Doki K, Homma M (2009). Co-administration of proton pump inhibitors delays elimination of plasma methotrexate in high-dose methotrexate therapy. Br J Clin Pharmacol.

[14] Deacon R, Lumb M, Perry J (1978). Selective inactivation of vitamin B12 in rats by nitrous oxide. Lancet.

[15] McColl KE (2009). Effect of proton pump inhibitors on vitamins and iron. Am J Gastroenterol.

[16] Joint Formulary Committee. British National Formulary (online) London: BMJ Group and Pharmaceutical Press. http://www.medicinescomplete.com. Accessed 5 Feb 2016

